# Supplementary material for: Cryo-EM structure of the Rous sarcoma virus octameric cleaved synaptic complex intasome
Source: Commun Biol. 2021 Mar 12;4:330. doi: 10.1038/s42003-021-01855-2 (PMC7955051; doi:10.1038/s42003-021-01855-2)
Supplement: Supplementary file 2 — Supplemental Information [file 42003_2021_1855_MOESM2_ESM.pdf]

Supplementary Information

Cryo-EM structure of the Rous sarcoma virus octameric cleaved synaptic complex intasome

Krishan K. Pandey[1,†,*], Sibes Bera[1,†], Ke Shi[2,†], Michael J. Rau[3], Amarachi V. Oleru[4], James A. J. Fitzpatrick[3,5,6], Alan N. Engelman[4,7], Hideki Aihara[2,*], and Duane P. Grandgenett[1,*]

[1] Department of Molecular Microbiology and Immunology, School of Medicine, Saint Louis University, St. Louis, MO 63104 USA

[2] Department of Biochemistry, Molecular Biology and Biophysics, University of Minnesota, Minneapolis, MN 55455 USA

[3] Washington University Center for Cellular Imaging, Washington University School of Medicine, St. Louis, MO 63110 USA

[4] Department of Cancer Immunology and Virology, Dana-Farber Cancer Institute, Boston, MA 02215 USA

[5] Departments of Cell Biology & Physiology and Neuroscience, Washington University in St. Louis, School of Medicine, St. Louis, MO 63110 USA

[6]Department of Biomedical Engineering, Washington University in St. Louis, St. Louis, MO 63130 USA

[7]Department of Medicine, Harvard Medical School, Boston, MA 02155 USA

[†]These authors contributed equally (co-first authors).

[*]Corresponding authors: krishan.pandey@health.slu.edu, aihar001@umn.edu, Duane.Grandgenett@health.slu.edu

This PDF file includes:

Supplementary Figures 1 to 12

Supplementary Fig. 1.

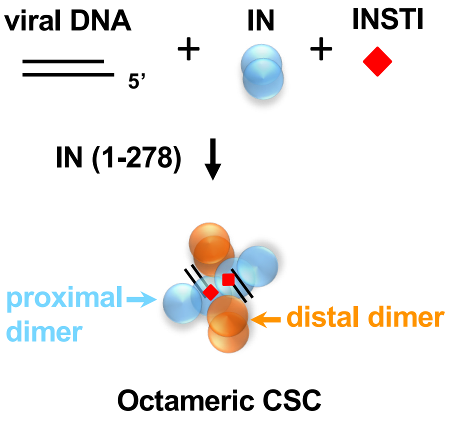

Supplementary Fig. 1. HIV-1 INSTI traps RSV intasome. The octameric RSV CSC can be assembled with different C-terminal truncated IN dimers in the presence of 3' OH recessed viral DNA (18 mer) and an INSTI. The octameric CSC used in this study is assembled by 4 IN dimers that end at K278 (IN 1-278). Wt RSV IN is 286 residues in length (See Supplemental Fig. 3).

Supplementary Fig. 2.

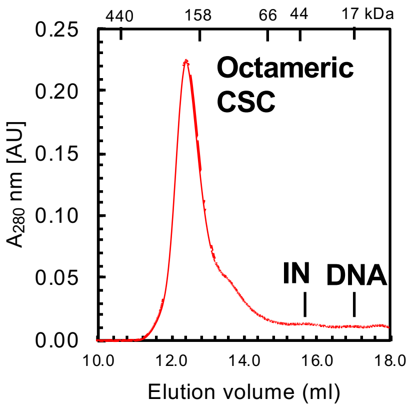

Supplementary Fig. 2. Purification of MK-2048 stabilized RSV octameric CSC for cryo-EM imaging. The assembled octameric CSC using IN 1-278 was injected onto a Superdex 200 Increase column (10x300). Absorbance at 280 nm was monitored. The positions of free IN and DNA are indicated. Size markers are indicated at the top.

Supplementary Fig. 3.

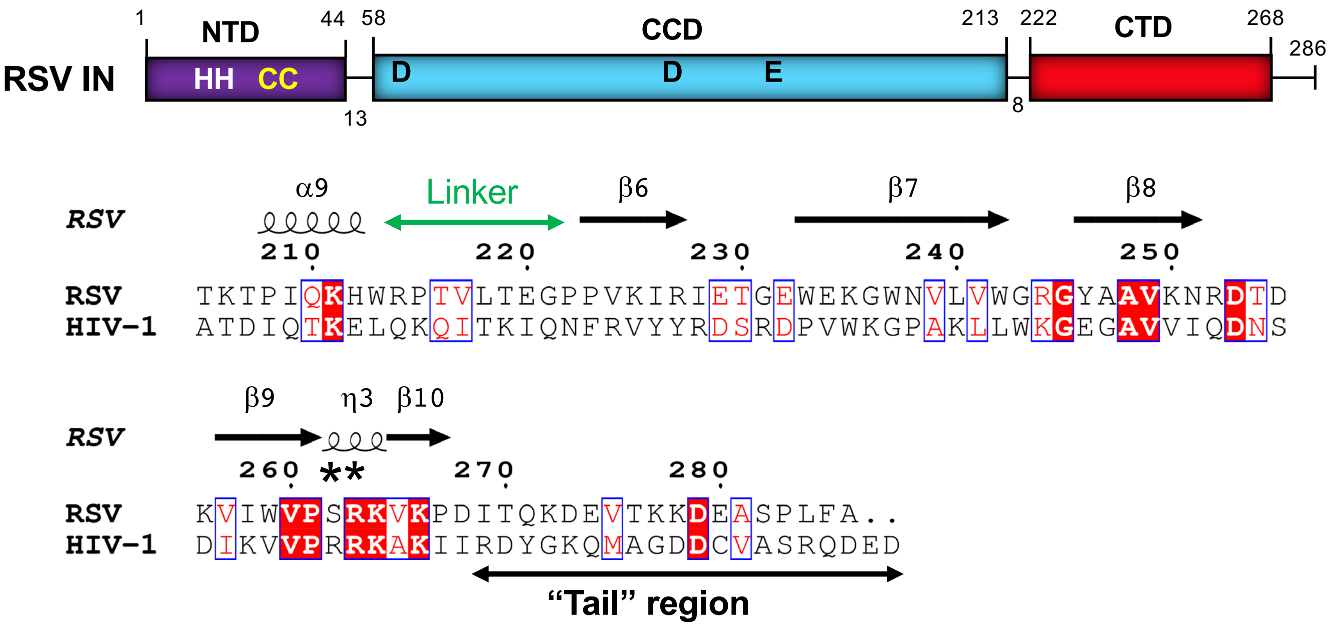

Supplementary Fig. 3. Amino acid sequence alignment of CTD and "tail" region of RSV (205-286 aa) with HIV-1 IN. The three-domain structure of RSV IN is shown at the top. The DDE catalytic triad comprises D64, D121 and E157 in the CCD domain. Secondary structures of RSV IN and the linker (green) between the CCD and CTD are identified. C-terminal truncations of the "tail region" and single missense changes in IN (marked by asterisk) were studied in infectivity assays and intasome assembly/activity assays. The RSV IN sequence (205-286 aa) was aligned in ClustalW and the alignment was used to generate the figure in ESPript3.0. The coloring scheme followed standard ESPript standards (Robert, X. and Gouet, P. (2014) "Deciphering key features in protein structures with the new ENDscript server". Nucl. Acids Res. 42(W1), W320-W324.)

Supplementary Fig. 4.

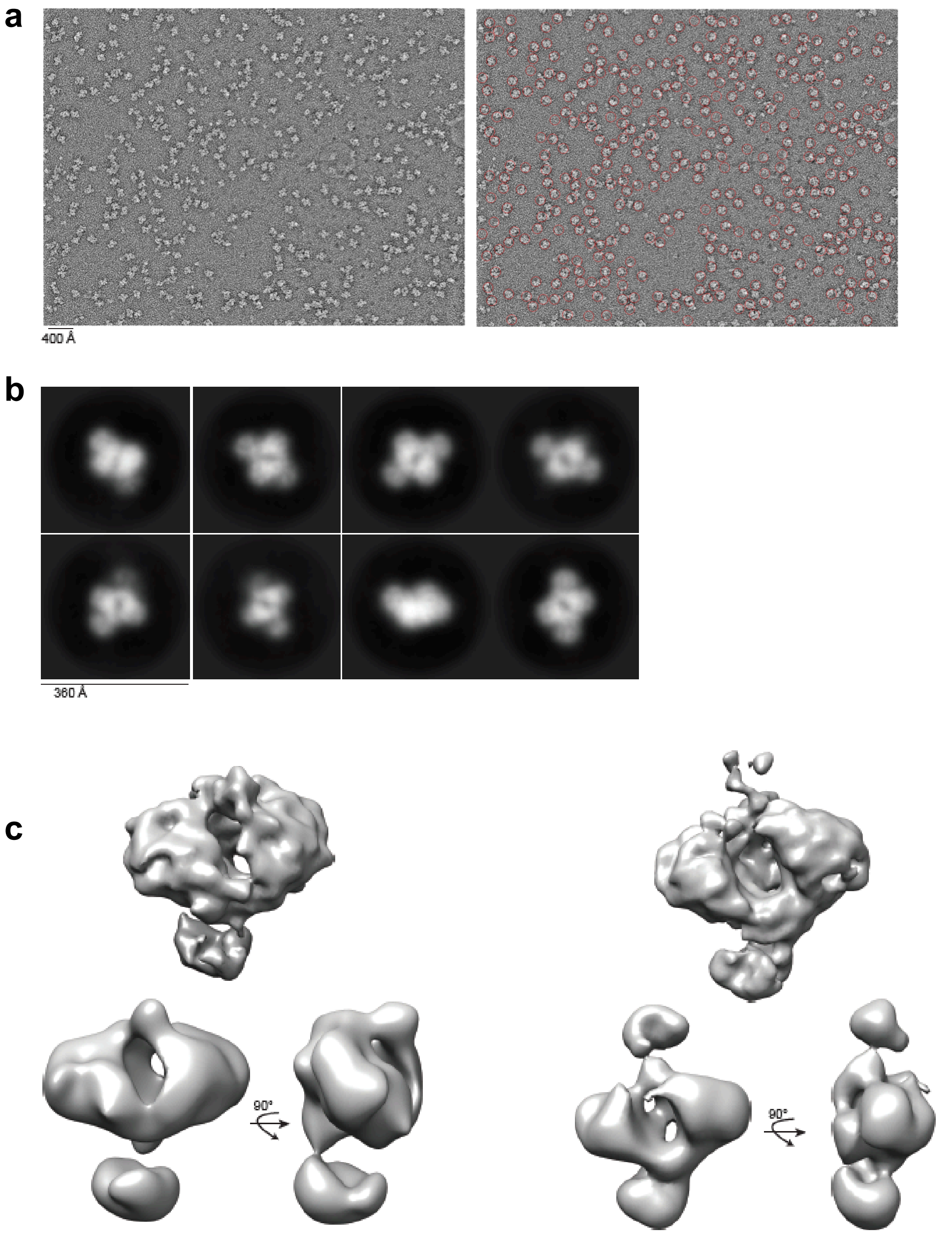

Supplementary Fig. 4. EM negative stain of octameric RSV CSC. **a.** A raw micrograph of the RSV CSC (left) and selected particles (red circles). **b.** Representative reference free 2D class-averages. **c.** 3D reconstructions of the RSV CSC in two classes and their refinement. Two distinct 3D classes show flexibility in binding of distal subunits.

Supplementary Fig. 5.

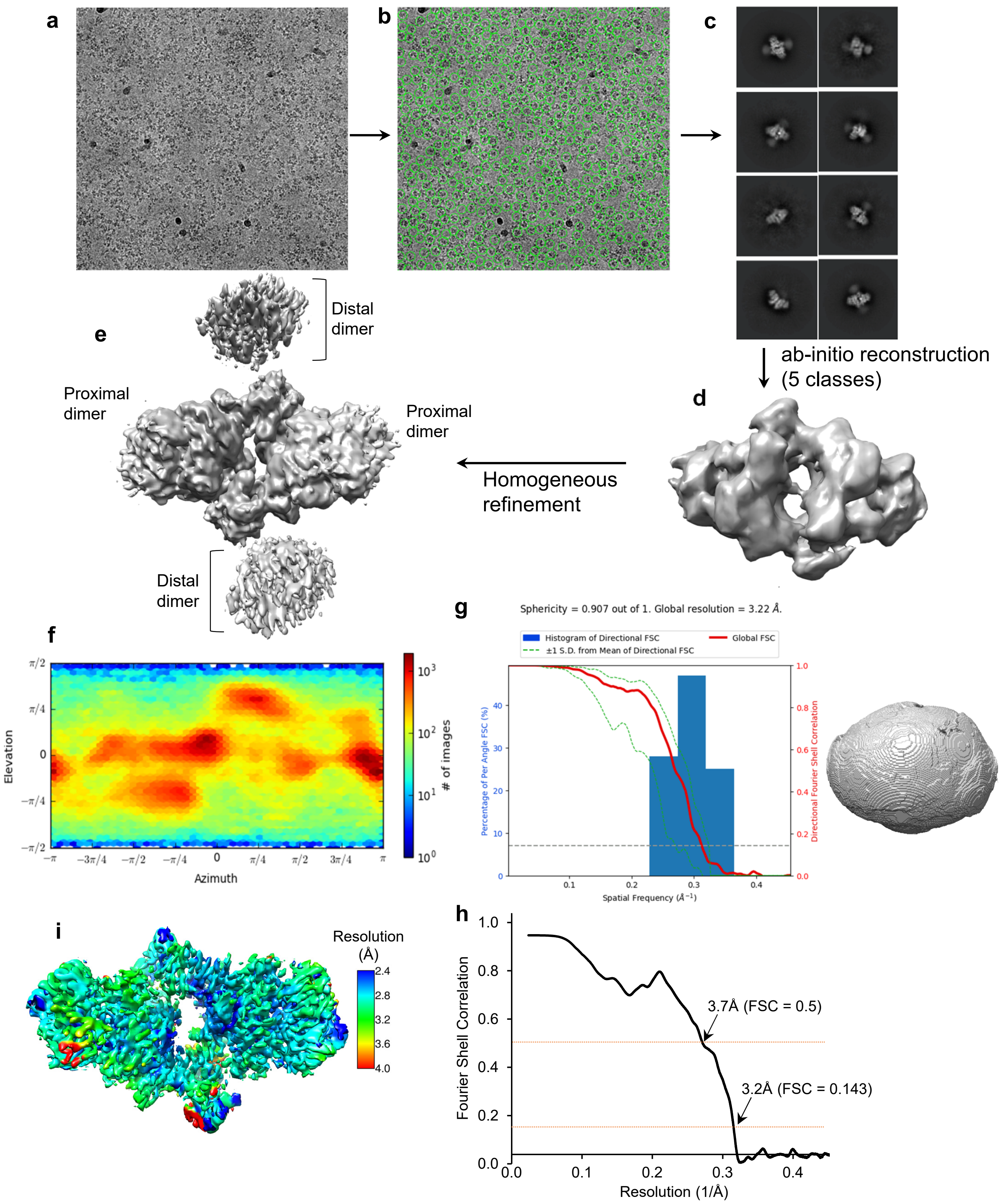

Supplementary Fig. 5. **Cryo-EM data processing workflow**. **a.** Representative cryo-EM micrograph. **b.** Automated particle (green circle) picking for the micrograph shown in **(a)**. **c.** Representative reference free 2-D class averages. **d.** The 3D class representing 456K particles produced by Ab-initio reconstruction. Only one of 5 ab-initio constructed map is shown. **e.** Homogeneous refinement of the 3D class shown in **(d)** results in 3D map at overall 3.21 Å resolution. **f.** Particle viewing directional distribution plot of the RSV CSC. **g.** Histogram of 1D FSC values overlaid with the average global FSC curve, shown alongside the binarized 3D FSC volume displayed at 0.143 threshold **h.** Model vs map FSC for the CIC region. **i.** Color coded local resolution map of the CIC region of RSV octameric CSC. The local resolution estimation was performed in cryoSPARC and displayed in Chimera.

Supplementary Fig. 6.

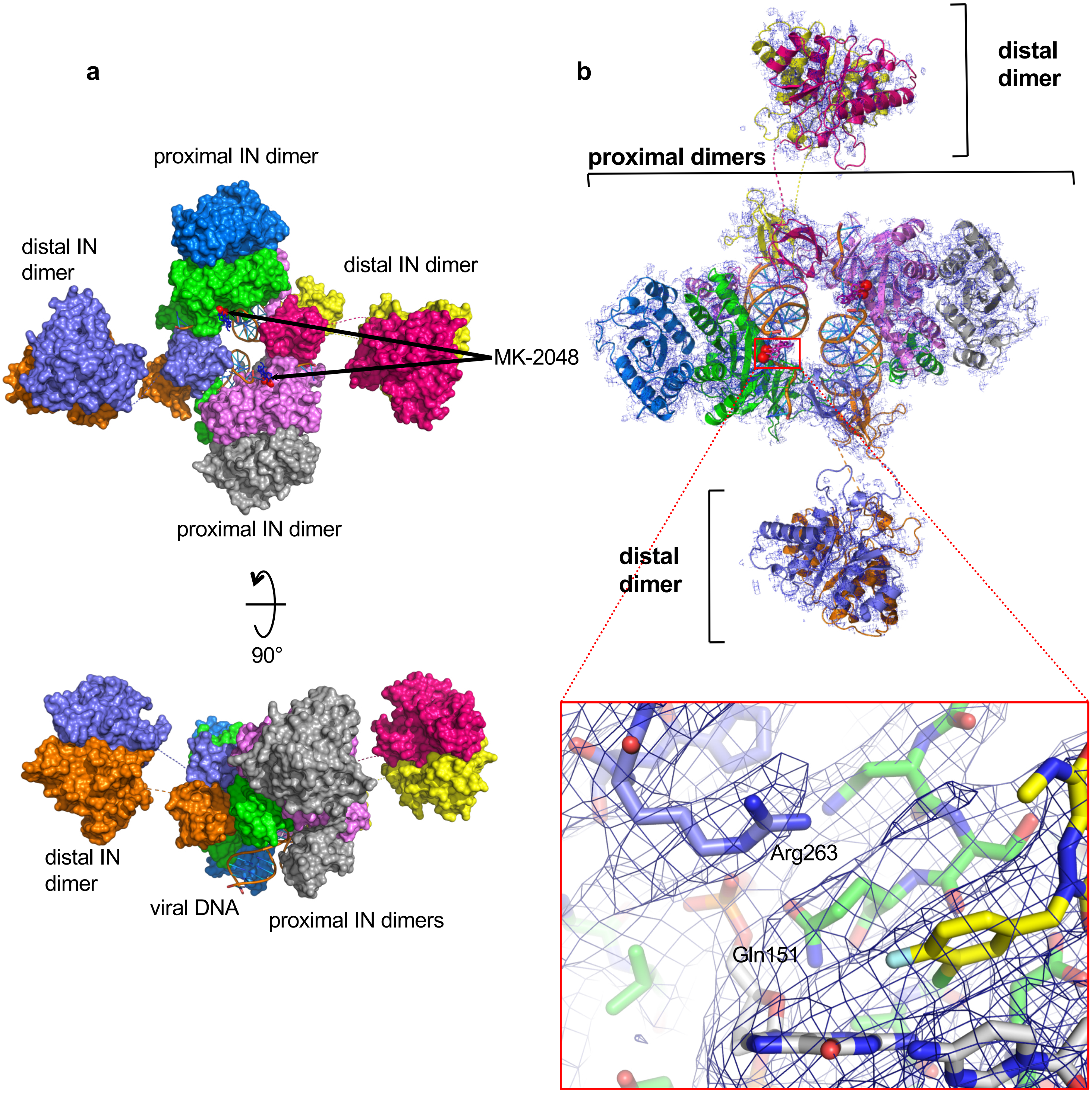

Supplementary Fig. 6. Cryo-EM structure of the RSV CSC trapped with MK-2048. a. Space filling representation of the CSC viewed down its pseudo 2-fold axis, with the 8 IN and viral DNA molecules colored differently. Two MK-2048 molecules in the active site of the catalytic IN protomers are shown in red. Disordered CCD-CTD linkers for the distal IN dimers are shown as dashed lines. **b.** Overlay of the atomic model on cryo-EM density. A zoomed view of Arg263-Gln151 interaction is shown.

Supplementary Fig. 7.

a. MK-2048

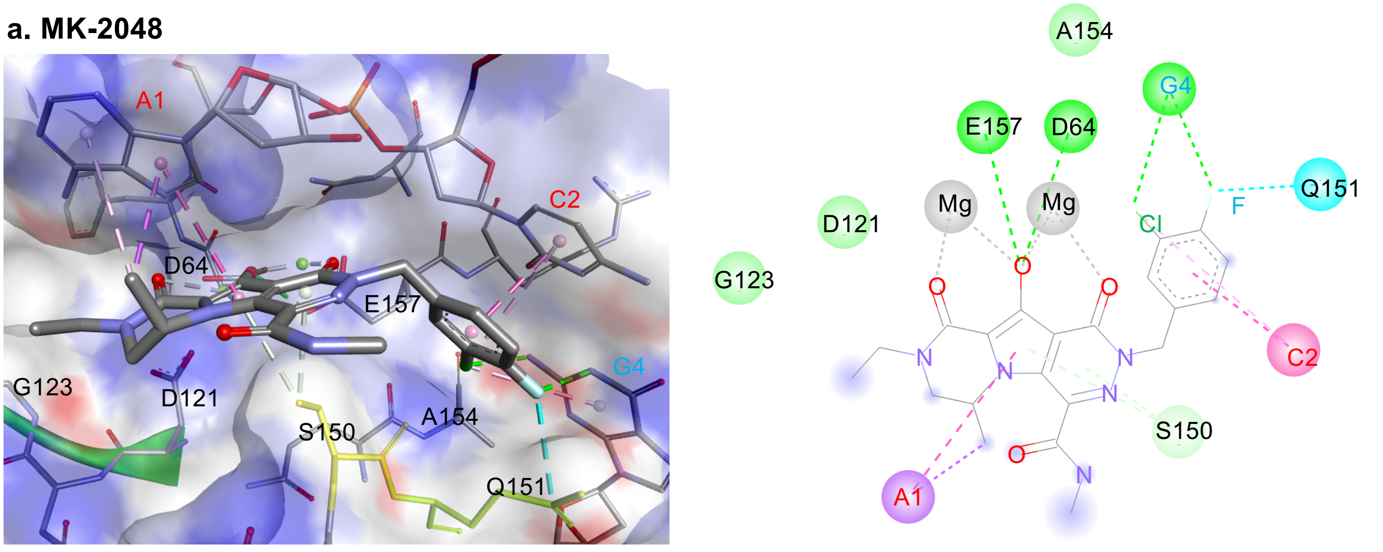

b. EVG

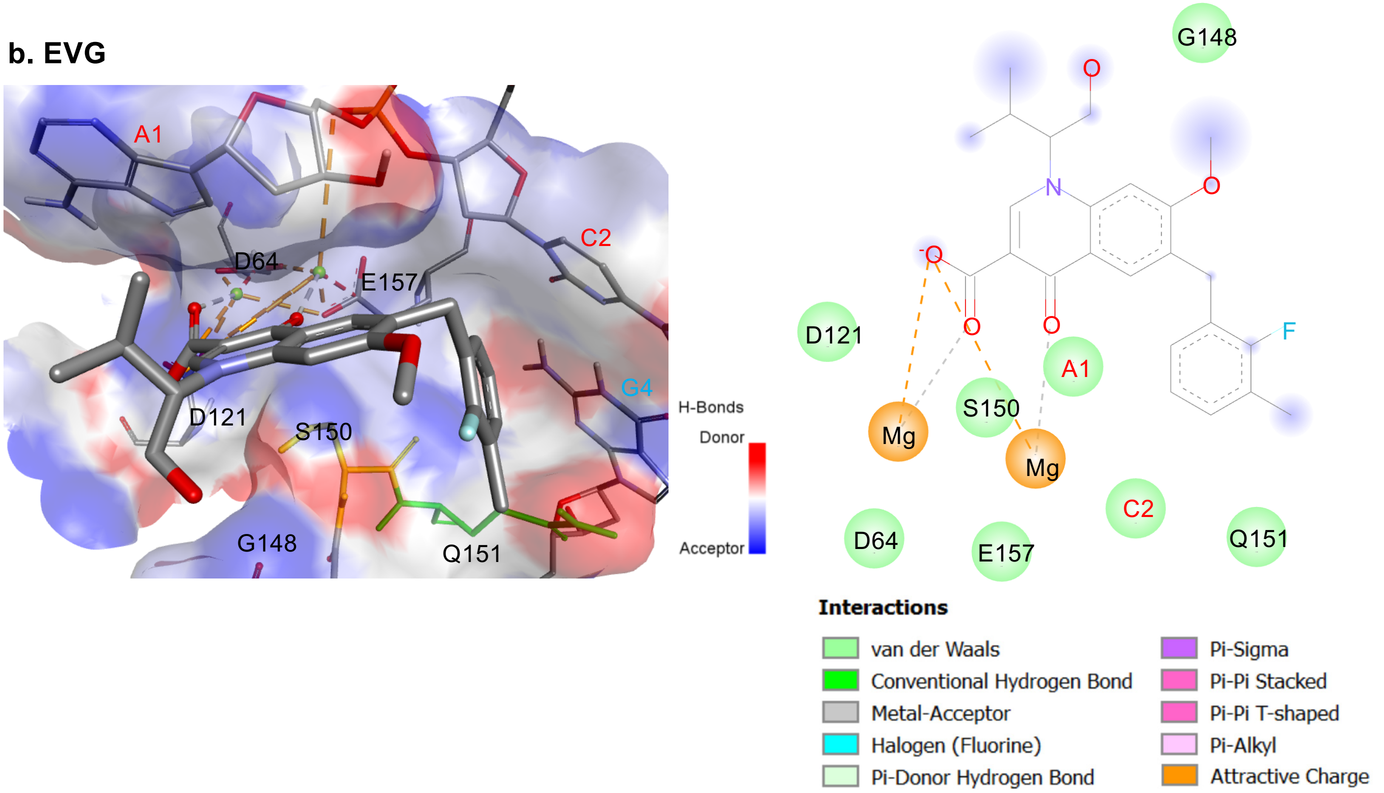

Supplementary Fig. 7. Interactions of INSTIs within the active site of octameric RSV CSC. a. Bound MK-2048 is shown in the RSV CSC. The viral DNA catalytic strand nucleotides A1 and C2 are labeled in red and non-catalytic strand G4 at the 4th position is labeled in cyan. A 2-D interaction diagram is shown on the right. Interactions are indicated. The purple halos in 2-D diagram demonstrates extents of solvent accessibility. **b.** INSTI EVG is docked into the IN active site within the CSC.

Supplementary Fig. 8.

a. MK-2048

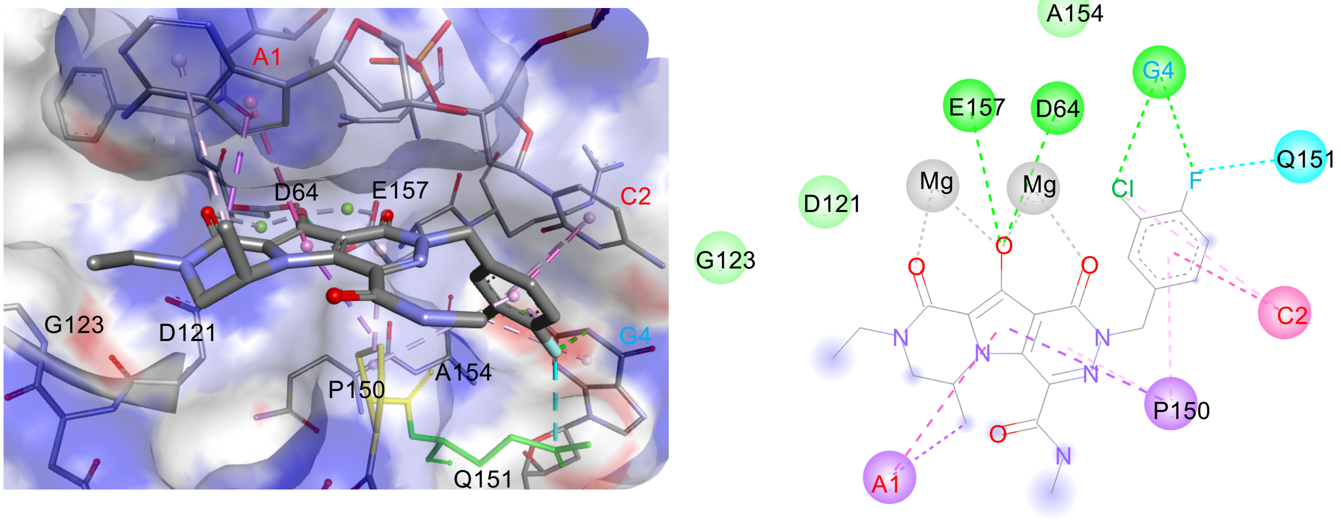

b. EVG

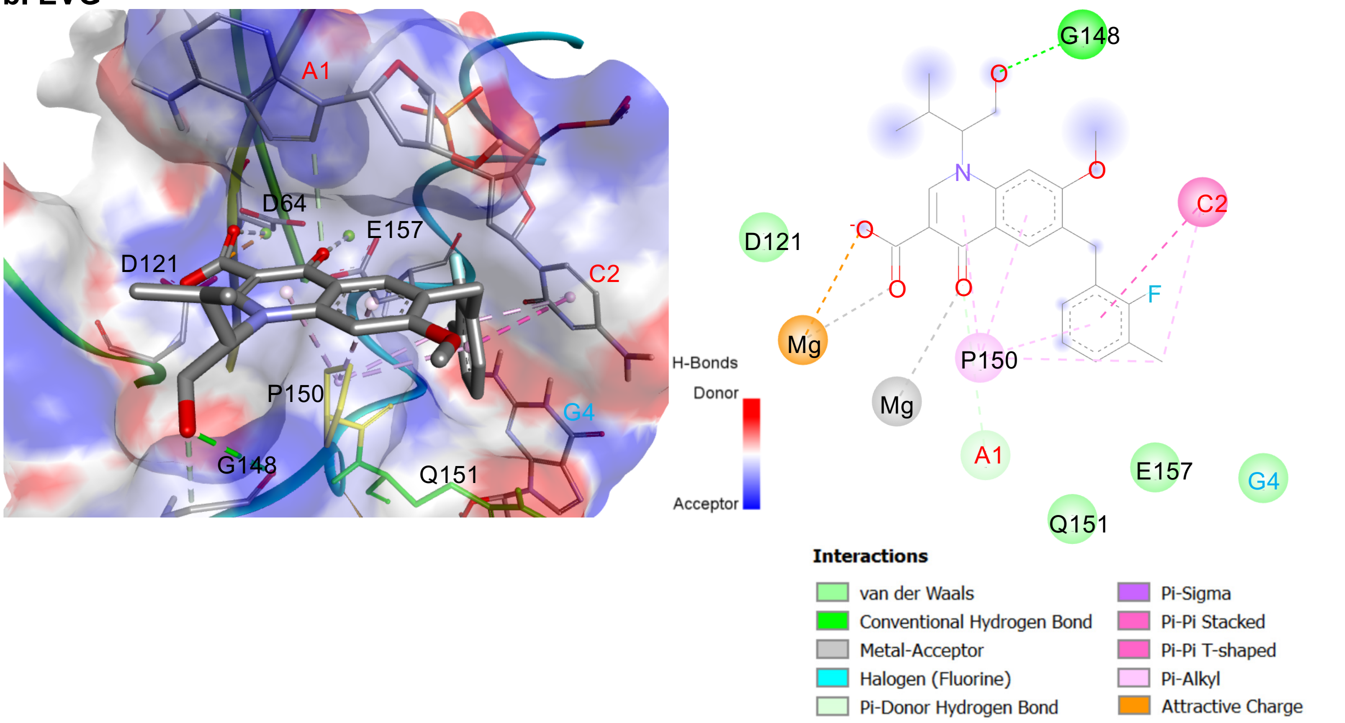

Supplementary Fig. 8. Docking of INSTIs in the active site of RSV IN (S150P) CSC. **a.** MK-2048 was docked into the octameric RSV CSC. The catalytic strand nucleotides A1 and C2 are labeled in red. The non-catalytic strand G4 is labeled in cyan. A two-dimension interaction diagram is shown on the right. The purple halos in the 2-D diagram demonstrates extents of solvent accessibility. Interactions are indicated. **b.** INSTI EVG is docked into the active site of IN S150P.

Supplementary Fig. 9.

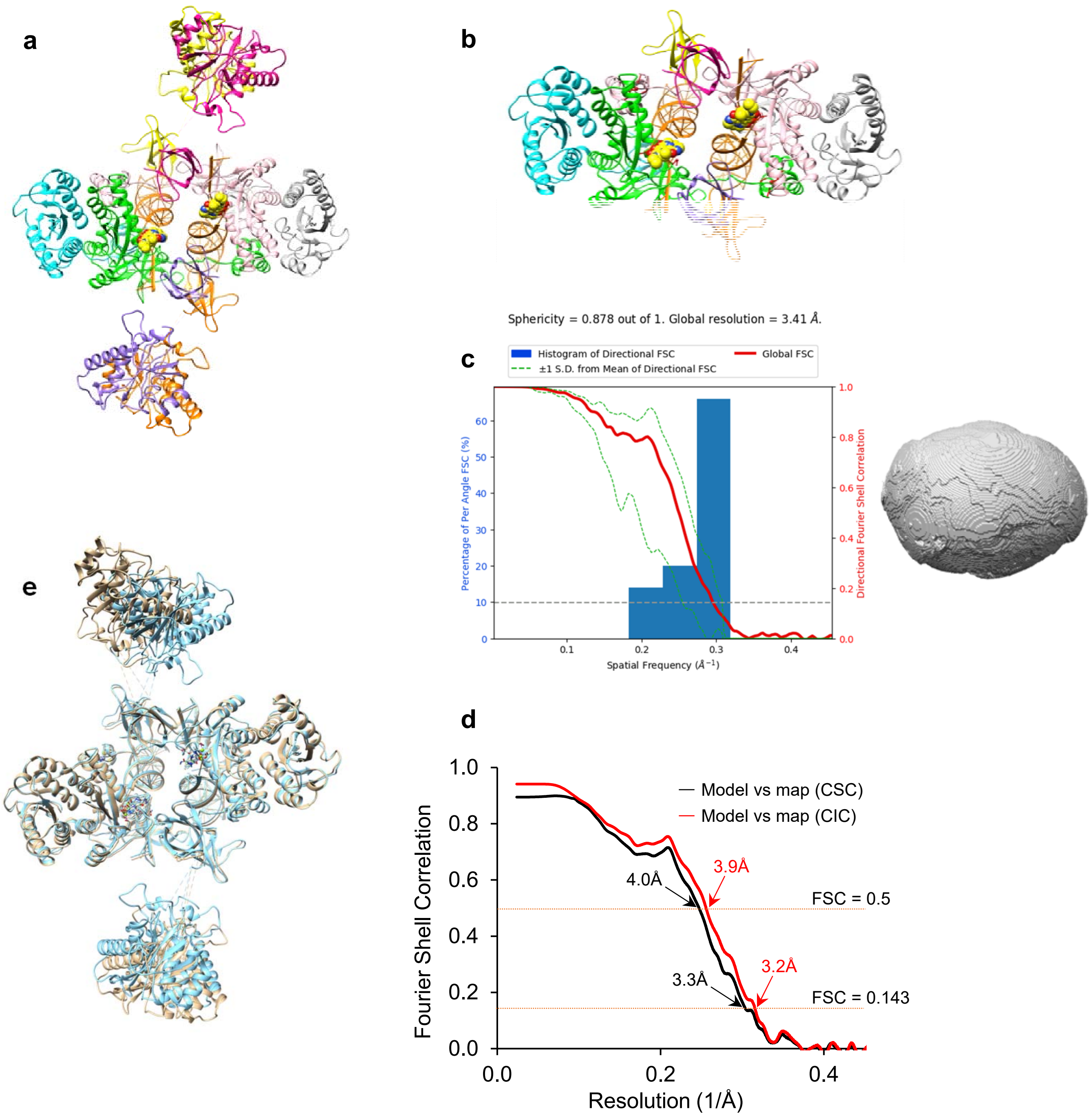

Supplementary Fig. 9. Cryo-EM structure of a cluster identified by 3-dimensional variability analysis. a. Cartoon representation of a CSC cluster depicting a particular orientation of distal dimer. **b.** Cartoon representation of the CIC region only. **c.** Histogram of 1D FSC values overlaid with the average global FSC curve, shown alongside the binarized 3D FSC volume displayed at 0.143 threshold. Reconstruction from this cluster showed relatively higher anisotropy and lower sphericity score as this structure represents a subset of particles in a particular conformation compared to the CSC. **d.** model vs map FSC for the CSC and CIC in cluster. **e.** Overlay of the cluster (cyan) with the overall CSC (tan) to demonstrate the conformational variability predominantly in distal subunits.

Supplementary Fig. 10.

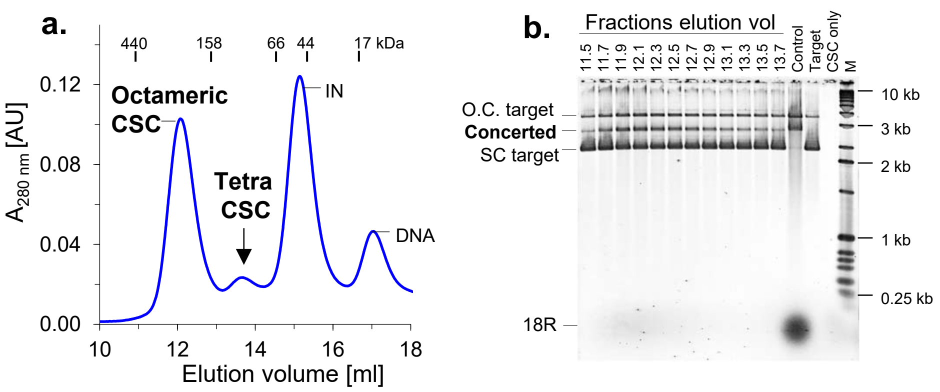

Supplementary Fig. 10. Octameric RSV CSC promotes concerted integration. a. The octameric CSC were assembled in the presence of IN, 3' OH recessed viral DNA and $MgSO_4$. The assembly was at 14ºC for 18 h and purification on Superdex 200 Increase (10x300) was at 4ºC. MK-2048 was omitted from the assembly and purification steps. The position of the octameric and tetrameric (tetra) CSC, free IN and DNA are shown. Size markers are indicated at the top. **b**. The elution volume for each fraction is indicated at the top. Each fraction (40 µl) was incubated for 30 min at 37ºC in the presence of supercoiled (s.c.) target DNA (5.7 nM). The reactions were stopped with EDTA and analyzed on 1.5 % agarose gels. The concerted integration product is marked. Recessed GU3 donor DNA (18 bp), open circular (o.c.) and s.c. target DNA are marked. The circular half-site integration reaction migrates with the o.c. target DNA. The control concerted reaction is purified RSV IN (1-278) with the donor (2 µM and 1 µM, respectively). Target DNA lane represents the target substrate by itself with IN (2 µM). The CSC only lane represents the peak fraction in the octameric CSC without target DNA. The gel was stained with SYBR Gold.

Supplementary Fig. 11.

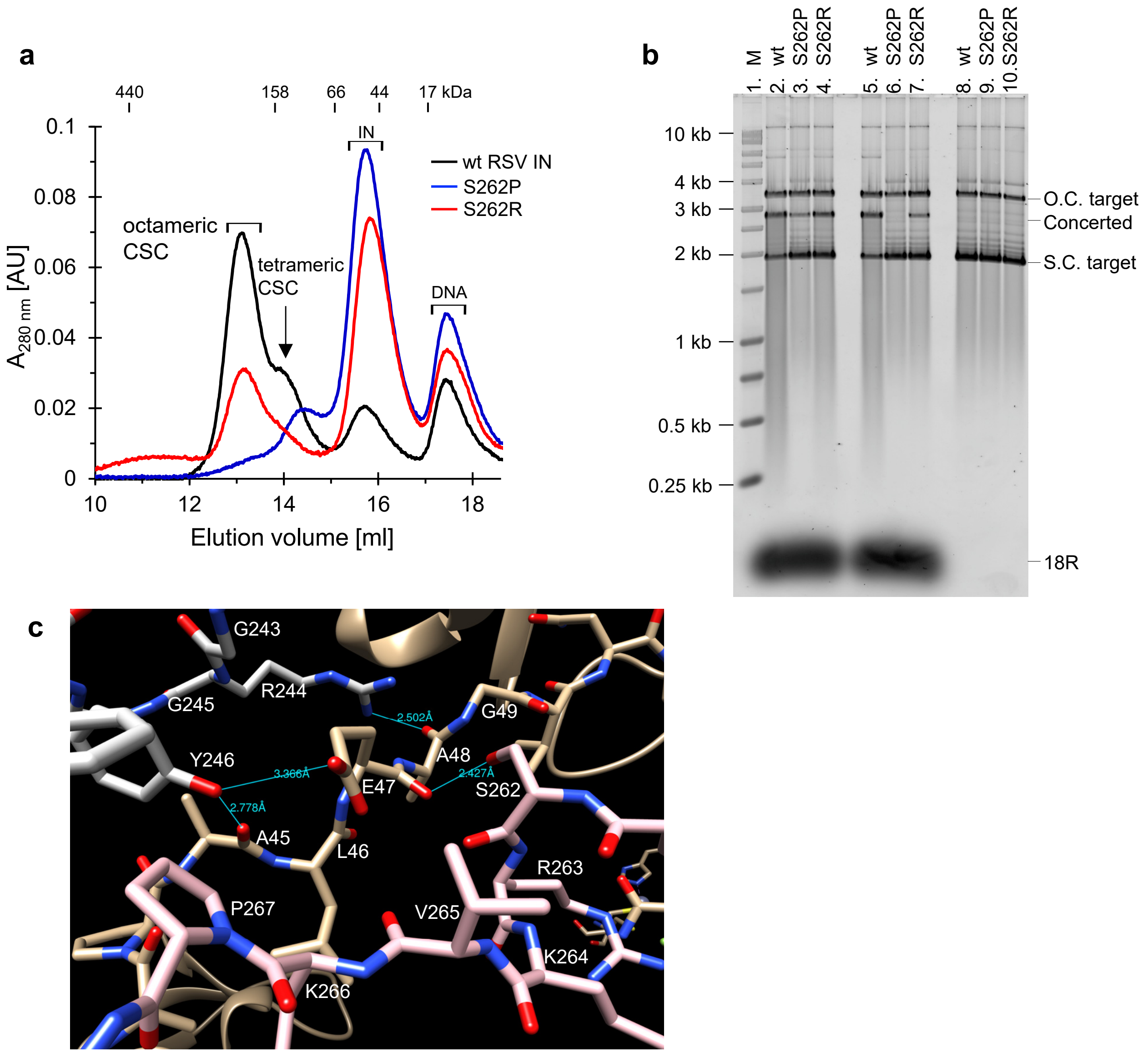

Supplementary Fig. 11. RSV S262 is critical for CSC assembly and concerted integration. a. CSCs were assembled with full-length RSV IN (1-286) or its mutants (S262P or S262R) at 18°C for 18 h with 18R GU3 and MK-2048 at 45,15, and 125 µM, respectively, were purified by SEC as described previously (15). The octameric and tetrameric CSC as well as free DNA and IN are indicated. **b.** Concerted integration assay was carried out with above mentioned IN (2 µM) and 18R (1 µM) at 125 mM NaCl (lanes 2-4 and 8-10) or 300 mM NaCl (lanes 5-7). Reactions in lanes 8-10 were carried out in absence of viral DNA to determine nonspecific DNA endonuclease activity in protein preparations. Lane 1 contains molecular weight marker ladder. **c.** CTD of distal INs (in pink and gray) make multiple interactions (shown are H-bonds) with inner catalytic subunit of proximal IN (tan color) in the NTD-CCD linker region to stabilize the assembly of the octameric CSC.

Supplementary Fig. 12.

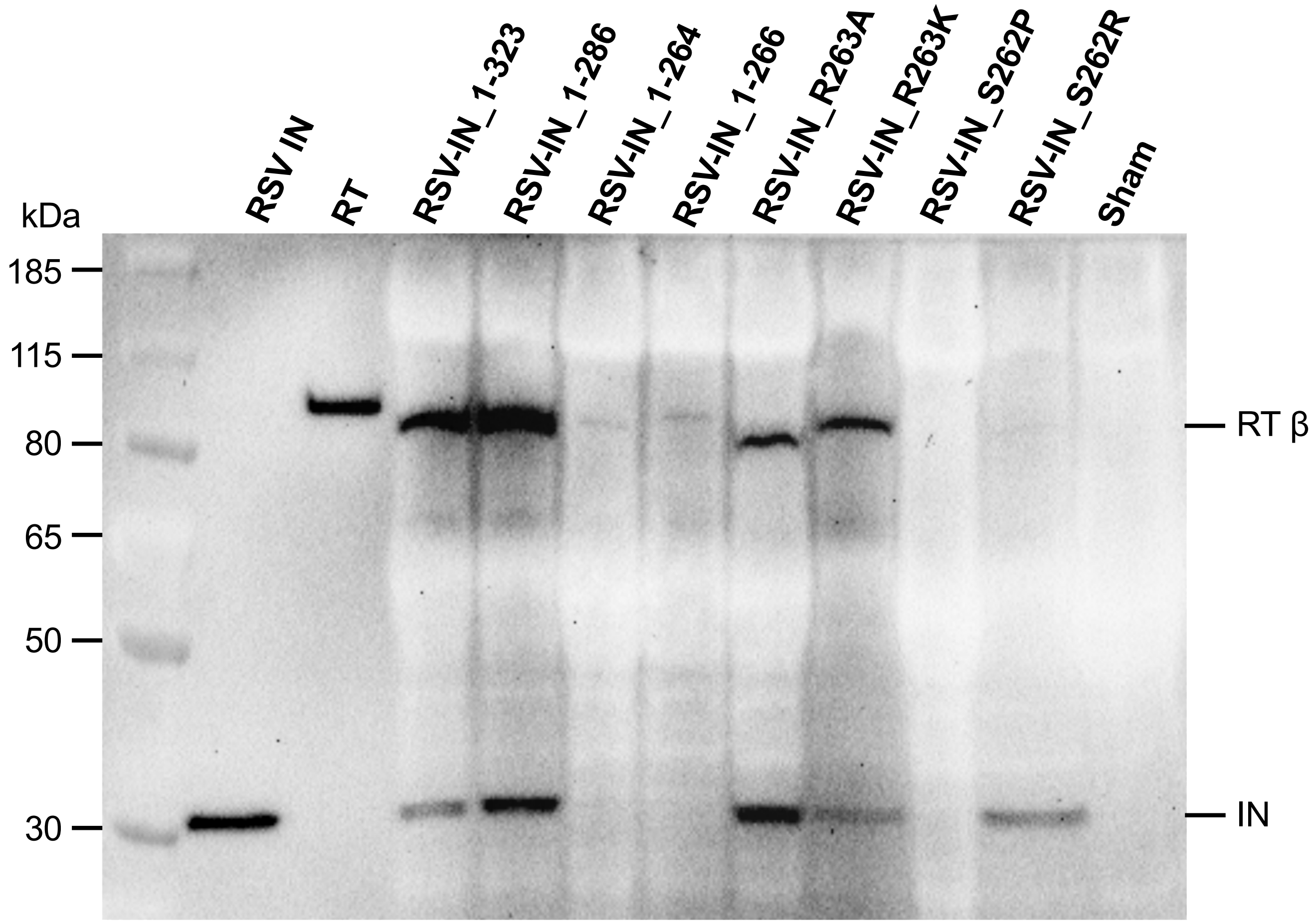

Supplementary Fig. 12. Expression of IN and the β polymerase subunits in virions. Equivalent quantities of viruses determined by virion-associated RT activity were subjected to SDS-gel analysis and probed by anti-IN antiserum produced against full-length AMV IN. From left to right, molecular weight markers, recombinant RSV IN (30 ng) and AMV αβ RT (0.4 U). The viruses are indicated above each lane. The Sham is the last lane. The RT β subunit (95 kDa) and IN (32 kDa) are identified on the right.
