# Supplementary material for: Cryo-EM structure of the Rous sarcoma virus octameric cleaved synaptic complex intasome
Source: Commun Biol. 2021 Mar 12;4:330. doi: 10.1038/s42003-021-01855-2 (PMC7955051; doi:10.1038/s42003-021-01855-2)
Supplement: Supplementary file 3 — Description of Additional Supplementary Files [file 42003_2021_1855_MOESM3_ESM.pdf]

Description of Additional Supplementary Files

File name: Supplementary Movie 1

Description: Dynamic flexibility of the RSV CSC intasome.

File name: Supplementary Data 1

Description: Source data used to prepare Fig. 5b and c.
