# Supplementary material for: Cryo-EM structure of the Rous sarcoma virus octameric cleaved synaptic complex intasome
Source: Commun Biol. 2021 Mar 12;4:330. doi: 10.1038/s42003-021-01855-2 (PMC7955051; doi:10.1038/s42003-021-01855-2)
Supplement: Supplementary file 6 — Reporting Summary [file 42003_2021_1855_MOESM6_ESM.pdf]

nature research

Corresponding author(s): Duane Grandgenett, Krishan Pandey, Hideki Aihara

Last updated by author(s): 12/08/2020

Reporting Summary

Nature Research wishes to improve the reproducibility of the work that we publish. This form provides structure for consistency and transparency in reporting. For further information on Nature Research policies, see our Editorial Policies and the Editorial Policy Checklist.

Statistics

For all statistical analyses, confirm that the following items are present in the figure legend, table legend, main text, or Methods section.

n/a	Confirmed	
☐	☒	The exact sample size (*n*) for each experimental group/condition, given as a discrete number and unit of measurement
☐	☒	A statement on whether measurements were taken from distinct samples or whether the same sample was measured repeatedly
☐	☒	The statistical test(s) used AND whether they are one- or two-sided<br>*Only common tests should be described solely by name; describe more complex techniques in the Methods section.*
☒	☐	A description of all covariates tested
☒	☐	A description of any assumptions or corrections, such as tests of normality and adjustment for multiple comparisons
☐	☒	A full description of the statistical parameters including central tendency (e.g. means) or other basic estimates (e.g. regression coefficient) AND variation (e.g. standard deviation) or associated estimates of uncertainty (e.g. confidence intervals)
☐	☒	For null hypothesis testing, the test statistic (e.g. F, t, r) with confidence intervals, effect sizes, degrees of freedom and P value noted<br>*Give P values as exact values whenever suitable.*
☒	☐	For Bayesian analysis, information on the choice of priors and Markov chain Monte Carlo settings
☒	☐	For hierarchical and complex designs, identification of the appropriate level for tests and full reporting of outcomes
☒	☐	Estimates of effect sizes (e.g. Cohen's d, Pearson's r), indicating how they were calculated

Our web collection on statistics for biologists contains articles on many of the points above.

Software and code

Policy information about availability of computer code

Data collection	cryo-EM data was collected by EPU 1.0
Data analysis	Cryo-EM data was analysed by RELION3.0, crypSPARC 2.12, 2.15, Chimera 1.13.1, pymol 2.3.1, PHENIX, Coot 0.9.<br>Other analysis was done in GraphPad Prism 8.

For manuscripts utilizing custom algorithms or software that are central to the research but not yet described in published literature, software must be made available to editors and reviewers. We strongly encourage code deposition in a community repository (e.g. GitHub). See the Nature Research guidelines for submitting code & software for further information.

Data

Policy information about availability of data

All manuscripts must include a data availability statement. This statement should provide the following information, where applicable:

- Accession codes, unique identifiers, or web links for publicly available datasets
- A list of figures that have associated raw data
- A description of any restrictions on data availability

The cryo-EM maps were deposited with the Electron Microscopy Data Bank (accession code EMDB-22400 and 23035) and the refined model with the Protein Data Bank (7JN3, 7KU7 and 7KUI). All other data are available upon request.

Field-specific reporting

Please select the one below that is the best fit for your research. If you are not sure, read the appropriate sections before making your selection.

☒ Life sciences ☐ Behavioural & social sciences ☐ Ecological, evolutionary & environmental sciences

For a reference copy of the document with all sections, see nature.com/documents/nr-reporting-summary-flat.pdf

Life sciences study design

All studies must disclose on these points even when the disclosure is negative.

Sample size	This work does not involve population study. No statistical methods were used to predetermine the sample size.
Data exclusions	No data was excluded from the analysis.
Replication	All attempts for replication of data were successful.
Randomization	Not relevant to our study. The study did not allocate samples in specific experimental groups.
Blinding	Blinding was not relevant for out study. Same investigator carried out the experiment and analyzed the data.

Reporting for specific materials, systems and methods

We require information from authors about some types of materials, experimental systems and methods used in many studies. Here, indicate whether each material, system or method listed is relevant to your study. If you are not sure if a list item applies to your research, read the appropriate section before selecting a response.

Materials & experimental systems

n/a	Involved in the study	
☐	☒	Antibodies
☐	☒	Eukaryotic cell lines
☒	☐	Palaeontology and archaeology
☒	☐	Animals and other organisms
☒	☐	Human research participants
☒	☐	Clinical data
☒	☐	Dual use research of concern

Methods

n/a	Involved in the study	
☒	☐	ChIP-seq
☒	☐	Flow cytometry
☒	☐	MRI-based neuroimaging

Antibodies

Antibodies used	Rabbit polyclonal anitbody used in Figure S12 was raised against a 14 amino acid peptide derived from sequences near the N-terminus of RSV integrase or the purified AMV full-length integrase eluted from an SDS-gel as described in Grandgenett et al., Virology 1983, 130: 257-262.
Validation	Grandgenett et al., Virology 1983, 130: 257-262.

Eukaryotic cell lines

Policy information about cell lines

Cell line source(s)	DF-1 cells were obtained from ATCC (CRL-12203)
Authentication	DF-1 cells were not authenticated beyond ATCC's own QC process
Mycoplasma contamination	DF-1 cells were verified in house as free from mycoplasma contamination
Commonly misidentified lines (See ICLAC register)	DF-1 is not listed in the ICLAC registry as a commonly misidentified line
